# Thermostability enhancement of *Escherichia coli* phytase by error-prone polymerase chain reaction (epPCR) and site-directed mutagenesis

**DOI:** 10.3389/fbioe.2023.1167530

**Published:** 2023-03-30

**Authors:** Hongguan Xing, Pingping Wang, Xing Yan, Yi Yang, Xinliang Li, Rui Liu, Zhihua Zhou

**Affiliations:** ^1^ School of Pharmacy, East China University of Science and Technology, Shanghai, China; ^2^ CAS-Key Laboratory of Synthetic Biology, CAS Center for Excellence in Molecular Plant Sciences, Institute of Plant Physiology and Ecology, Chinese Academy of Sciences, Shanghai, China; ^3^ CJ Youtell (Shanghai) Biotech Co., Ltd., Shanghai, China

**Keywords:** phytase, thermostability, error-prone PCR, directed evolution, disulfide bond

## Abstract

Phytase efficiently hydrolyzes phytate to phosphate; thus, it is widely used to increase phosphorus availability in animal feeds and reduce phosphorus pollution through excretion. Phytase is easily inactivated during feed pelleting at high temperature, and sufficient thermostability of phytase is essential for industrial applications. In this study, directed evolution was performed to enhance phytase thermostability. Variants were initially expressed in *Escherichia coli* BL21 for screening, then in *Pichia pastoris* for characterization. Over 19,000 clones were generated from an error-prone Polymerase Chain Reaction (epPCR) library; 5 mutants (G10, D7, E3, F8, and F9) were obtained with approximately 9.6%, 10.6%, 11.5%, 11.6%, and 12.2% higher residual activities than the parent after treatment at 99°C for 60 min. Three of these mutants, D7, E3, and F8, exhibited 79.8%, 73.2%, and 92.6% increases in catalytic efficiency (*kcat/Km*), respectively. In addition, the specific activities of D7, E3, and F8 were 2.33-, 1.98-, and 2.02-fold higher than parental phytase; they were also higher than the activities of all known thermostable phytases. Sequence analysis revealed that all mutants were substituted at residue 75 and was confirmed that the substitution of cysteine at position 75 was the main contribution to the improvement of thermostability of mutants by saturation mutagenesis, indicating that this amino acid is crucial for the stability and catalytic efficiency of phytase. Docking structure analysis revealed that substitution of the C75 residue allowed the mutants to form additional hydrogen bonds in the active pocket, thereby facilitating binding to the substrate. In addition, we confirmed that the intrinsic C77-C108 disulfide bond in *E. coli* phytase is detrimental to its stability.

## 1 Introduction

Phytate is the major form of phosphorus stored in cereal grains and legumes used in commercial animal feeds (Reddy et al., 1982; Tahir et al., 2012). However, monogastric animals cannot utilize phosphorous in phytate because they lack the necessary digestive enzymes. Undigested or unabsorbed phytate is excreted in the form of inorganic phosphorus, which has caused multiple serious environmental pollution issues (Li et al., 2022). In addition, because of its negative charge, phytate often complexes with metal ions and proteins to form anti-nutritive insoluble complexes (Khajali and Slominski, 2012). Phytases are enzymes that catalyze the stepwise hydrolysis of phytate into myoinositol and inorganic phosphate; they have been widely applied in animal feeds to increase phosphorus availability, decrease the anti-nutritional effects of phytate, and reduce phosphorus pollution (Dalsgaard et al., 2023; Selle et al., 2023).

Since the first description of phytase in 1907, numerous wild-type and engineered enzymes derived from plants, animals, and microorganisms have been reported (Mullaney et al., 2000). Among these enzymes, microbial phytases of fungal and bacterial origin are the most promising candidates for biotechnological application on a commercial scale in the feed market (Jain et al., 2016). *Escherichia coli* AppA phytase has high catalytic efficiency and specific activity; it has thus attracted substantial research interest (Vohra and Satyanarayana, 2003). However, the use of phytase as a feed enzyme requires tolerance of high temperatures because animal feed is commonly pelleted to ensure that animals have a balanced diet; the industrial pelleting process also facilitates storage of the enzyme-containing product. During the pelleting process, temperatures may temporarily reach 90°C and cause phytase inactivation (Vohra and Satyanarayana, 2003). Thus, industrial feed production requires the identification of phytases with high thermostability and the ability to tolerate the transient high-temperature step in the pelleting procedure.

Directed evolution has proven to be a powerful strategy for protein engineering, especially with regard to improvements of protein activity and thermostability (Ruan et al., 2022; Su et al., 2022). This process involves the generation of a vast mutant library of the gene of interest through random mutagenesis techniques (e.g., error-prone Polymerase Chain Reaction [epPCR] or DNA shuffling), which are then screened to identify mutants that enhance enzymatic performance in non-natural environments (Athavale et al., 2022). The efficient generation of random mutations in the target gene is an important step in directed evolution. Because it can reduce the fidelity of Taq DNA polymerase during DNA synthesis and thus promote random substitutions in template sequences (Cadwell and Joyce, 1992), epPCR has been widely and successfully used in directed evolution. Kim et al. employed epPCR to improve AppA2 phytase thermostability; compared with the control enzyme, two variants showed >20% improvement in thermostability (80°C for 10 min), along with 6°C-7°C increases in melting temperature (Kim and Lei, 2008). Additionally, epPCR and rational design reportedly improved xylanase thermostability, such that the optimal variant had an 820-fold higher melting temperature than the wild-type enzyme at 70°C (Xing et al., 2021).

In recent years, directed evolution has considerably improved phytase thermostability; numerous engineered phytases have been applied in the feed and fuel industries under high-temperature conditions (Mrudula Vasudevan et al., 2019). However, it remains challenging to balance the thermal stability and specific activity of thermostable phytases. For example, the *Bacillus amyloliquefaciens*-derived phytase US573 and the *Aspergillus niger*-derived phytase phyA have excellent heat resistance, with residual activities of 50% and 37%, respectively, after treatment at 100°C for 10 min; however, their specific activities are only 27 U/mg and 72 U/mg (Ushasree et al., 2014; Boukhris et al., 2015). A thermostable mutant of *E. coli*-derived phytase (K74N/K75Q/G76T) has high specific activity (2370.4 ± 135.7 U/mg) but only demonstrated 9.7% residual activity after treatment at 80°C for 5 min (Wu et al., 2014). Because the heating process used in feed production requires that the additive phytase has high thermal stability and retains considerable activity, there is a need to identify new phytases with high thermal stability and activity. In this study, we sought to improve the industrial features of *E. coli* AppA phytase for feed pelleting; thus, we used a modified epPCR protocol to generate a mutant library, followed by directed evolution to enhance thermostability while maintaining high enzyme activity. In addition, we successfully expressed *E. coli* AppA phytase in *Pichia pastoris* with a high yield; our findings provide a firm foundation for future commercial applications.

## 2 Materials and methods

### 2.1 Materials

The gene encoding parental *E. coli* AppA phytase was synthesized by Sangon Biotech (Shanghai, China). Random mutagenesis kits for epPCR were obtained from Agilent (California, United States). The AxyPrep plasmid miniprep and AxyPrep DNA gel extraction kits were purchased from Axygen (California, United States). The One Step Cloning Kit was obtained from Vazyme (Nanjing, China). All other chemicals and reagents were purchased from Sangon (Shanghai, China) and were of analytical grade. The expression vector pET28a in *E. coli* was obtained from Novagen (Madison, Wisconsin, United States). The expression vector pPICZαA (Invitrogen, United States ) in *P. pastoris* was obtained from Invitrogen (San Diego, United States). *E. coli* BL21 (DE3) was used as the host for screening, while *P. pastoris* was used for recombinant protein expression and characterization.

### 2.2 Methods

#### 2.2.1 Construction of epPCR random mutation library and site-directed mutagenesis

epPCR was performed using the pET28a plasmid as a template; this plasmid contained the parental phytase gene. The 50-µL reaction mixture contained 33.7 µL of double-distilled water, 5 µL of 10× Mutazyme II reaction buffer, 1 µL of 40 mM dNTP mix (final concentration of 200 µM per dNTP), 2 µL of primer mix, 1 µL of Mutazyme II DNA polymerase (2.5 U/µL), and 7.3 µL of template (500 ng of total DNA). The polymerase chain reaction (PCR) protocol comprised 30 cycles of 95°C for 2 min, 55°C for 30 s, and 72°C for 1.3 min. The PCR product was gel-purified and ligated to the *BamHI* and *HindIII* multiple cloning sites of the pET28a vector. The ligation product was transformed into *E. coli* BL21. The transformed cells were incubated on lysogeny broth plates containing kanamycin at 37°C for 12 h.

#### 2.2.2 Site-directed mutagenesis and site-saturations mutagenesis

Site-directed and site-saturated mutagenesis were performed following the instruction of One Step Cloning Kit (Vazyme, China). The plasmid pET28a containing parental phytase gene was used as the template to produce the mutant phytases. The amplification fragments were digested using DpnI at 37°C for 1.5 h and transformed to Top10 for screening.

#### 2.2.3 High-throughput screening for thermostable variants

Individual colonies of transformants were transferred into 96-well plates that contained 400 µL of lysogeny broth medium per well. The 96-well plates were cultured at 37°C with shaking at 180 rpm for 12 h. Then, the culture was transferred to another 96-well plate that contained 500 μL of fresh lysogeny broth medium with a final isopropyl β-D-1-thiogalactopyranoside (IPTG) concentration of 0.001 mM. After induction at 37°C for 6 h, all mutants were heat treated at 80°C for 20 min. Subsequently, 4 µL of enzyme from the heat-treated culture supernatants of each mutant were transferred to a new 96-well plate and mixed with 80 μL of sodium phytate substrate solution (7.5 mM) and 36 μL of acetate buffer (pH 5.5). After incubation at 37°C for 10 min, the reaction was stopped by the addition of 80 μL of stop solution. The residual activity of each mutant was detected based on absorbance at 415 nm. Clones with higher residual activities, compared with the parent, were regarded as potential positive clones.

#### 2.2.4 Protein expression and purification in *P. pastoris*

The parent and mutant genes of phytase without the native signal peptide were inserted into the expression vector pPICZαA (Invitrogen, United States) at the *EcoRI* site under the control of the AOX1 promoter. The resulting plasmid was linearized using the restriction enzyme *SacI* and transformed into the host strain KM71 through electroporation (Bio-Rad, United States). The transformed cells were incubated on yeast extract, peptone, dextrose (YPD) plates containing zeocin at 30°C for 3 days, then verified by sequencing. Selected colonies of the transformants were placed into 4-mL YPD medium in test tubes and incubated at 30°C overnight. Then, 300 μL of the culture suspension were transferred into 300 mL of buffered glycerol complex (BMGY) medium and incubated at 30°C. After the optical density at 600 nm (OD_600_) reached 4–6, the cells were collected by centrifugation and resuspended in 30 mL of buffered methanol complex (BMMY) medium, in which the 1% glycerol of BMGY had been replaced with 1% methanol, for phytase expression. To assess the expression of parent and mutant enzymes, 10 µL of culture supernatant were subjected to sodium dodecyl sulfate–polyacrylamide gel electrophoresis. For purification of the parental and mutant enzymes, 30 mL of culture supernatant were collected *via* centrifugation and transferred to a His-Tag Ni-affinity column (Qiagen, Hilden, Germany) that had been equilibrated with Tris-HCL buffer (pH 8.0). Target proteins were eluted with a linear imidazole gradient from 0 to 100 mM in Tris-HCL buffer. Protein concentrations were determined using the Bradford assay.

#### 2.2.5 Phytase activity assay

Phytase-specific activity was measured using a spectrophotometric assay that detected the amount of free phosphate liberated from sodium phytate in 0.25 M CH_3_COONa at pH 5.5. For this measurement, 4 µL of enzyme were added to 116 µL of assay mix (80 µL of 7.5 mM sodium phytate and 36 µL of 0.25 M CH_3_COONa, pH 5.5) in a 96-well plate. The mixture was incubated at 37°C for 30 min. The reaction was stopped by the addition of 80 µL of coloration reagent (HNO3, (NH_4_)6Mo7O_24_·4H_2_O, and NH_4_VO_3_ at a ratio of 2:1:1). The amount of inorganic phosphate released was determined through the measurement of spectrophotometric absorbance at 415 nm. One unit of phytase activity was defined as the amount of enzyme required to liberate 1 µmol of inorganic phosphate from 5.0 mM phytate over 1 min at 37°C and pH 5.5. The kinetic parameters were evaluated using the Lineweaver–Burk method.

#### 2.2.6 Thermostability assay

Purified enzymes of each mutant type were diluted in 0.25 M sodium acetate buffer (pH 5.5) and incubated at 99°C for various lengths of time. Each enzyme was placed on ice for 10 min immediately after heat treatment and the residual activity mutant was tested at 37°C, as previously described. The initial activity (un-heated) measured at 37°C was regarded as 100% activity; residual activity was determined as a proportion of the initial activity.

#### 2.2.7 Homology modeling and docking simulations of parent and mutant phytase

The AppA phytase from *E. coli* (Protein Data Bank ID: 1DKP) was selected as template because it shares 97% sequence homology with parental phytase. The tertiary structures of mutant phytases were modeled using the SWISS-MODEL server (https://swissmodel.expasy.org/) and used as the receptor for docking simulations. Receptor-ligand docking simulations were conducted using the AutoDock Tools 1.5.6 platform (Cosconati et al., 2010). A diagram of the 3D structure of phytase was constructed using PyMOL software.

#### 2.2.8 Statistical analysis

All data are presented as means ± standard deviations of three independent replicates. Student’s t-test was used for statistical analysis in Microsoft Excel (Microsoft Corporation, Redmond, WA, United States). *p*-values <0.05 were considered indicative of statistical significance.

## 3 Results and discussion

### 3.1 Construction and screening of the random mutagenesis library of *E. coli* AppA phytase

**FIGURE 1:**
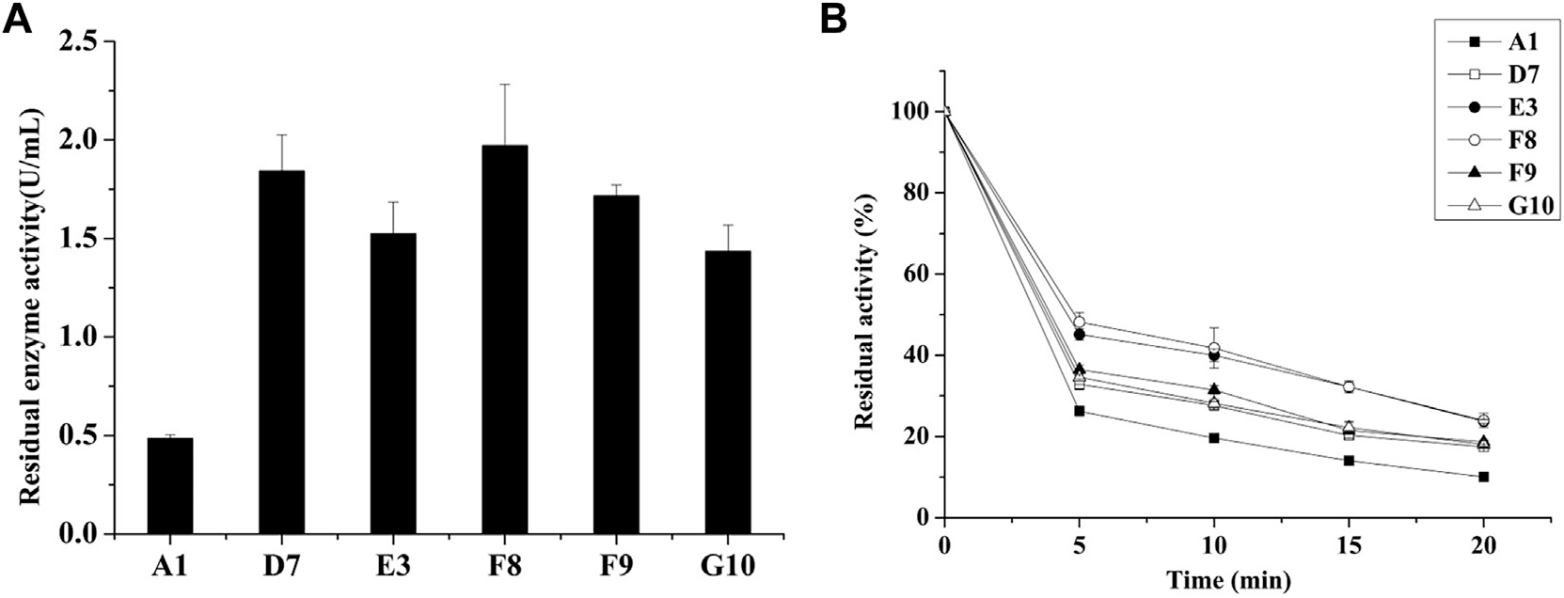
Screening of thermostable mutants in the *E. coli* BL21 host. **(A)** Residual activities of parent and mutants after treatment at 80°C for 20 min in 96-well plates. **(B)** Expression of thermostable mutants in *E. coli* and determination of thermostability after treatment at 80°C.

Directed evolution combined with *in vitro* high-throughput screening is a powerful method for the generation of mutant proteins with improved functional properties or the achievement of tolerance to strict reaction conditions (Huang et al., 2023). High genetic diversity in a target gene can be generated by epPCR through random base substitutions during DNA replication; this approach is widely used in experimental analyses. Recently, a modified epPCR method with reduced mutational bias from PCR has been reported (Qian et al., 2015). To increase the thermostability of *E. coli* AppA phytase, we initially generated a random mutation library for AppA phytase using this modified epPCR protocol. The first round of screening was performed on a 96-well plate; we selected mutants that exhibited greater residual activity after heating at 80°C for 20 min, compared with the parent. We screened nearly 19,000 clones and obtained 5 mutants with improved residual enzyme activity (Figure 1). These mutants, designated D7, E3, F8, F9, and G10, retained enzymatic activities of 1.84, 1.52, 1.97, 1.71, and 1.43 U/mL, respectively, after treatment at 80°C for 20 min; these values were significantly higher than the activity of parent (A1, 0.48 U/mL, *p* < 0.05). To further characterize the thermostabilities of the five mutants, their residual enzyme activities were tested after treatment at 80°C for various lengths of time (Figure 1B). The mutant strains D7, E3, F8, F9, and G10 retained approximately 19.3%, 23.7%, 24.0%, 18.6%, and 18.2% enzyme activity, respectively, after treatment at 80°C for 20 min, it is about 2-folds than those of parent (10.0%). Analysis of residue substitution sites in the mutants (Table 1) showed that all variants had substitutions at C75, suggesting that C75 had a central role in the enhanced thermal stability of the mutants.

**TABLE 1:** Substituted residues of mutants.

Mutants	Substitute residues
D7	C75Y/R404H
E3	C75Y
F8	C75S/V132L
F9	C75W/A84T/N176D
G10	C75S/I300F

### 3.2 High expression of phytase in *P. pastoris*

**FIGURE 2:**
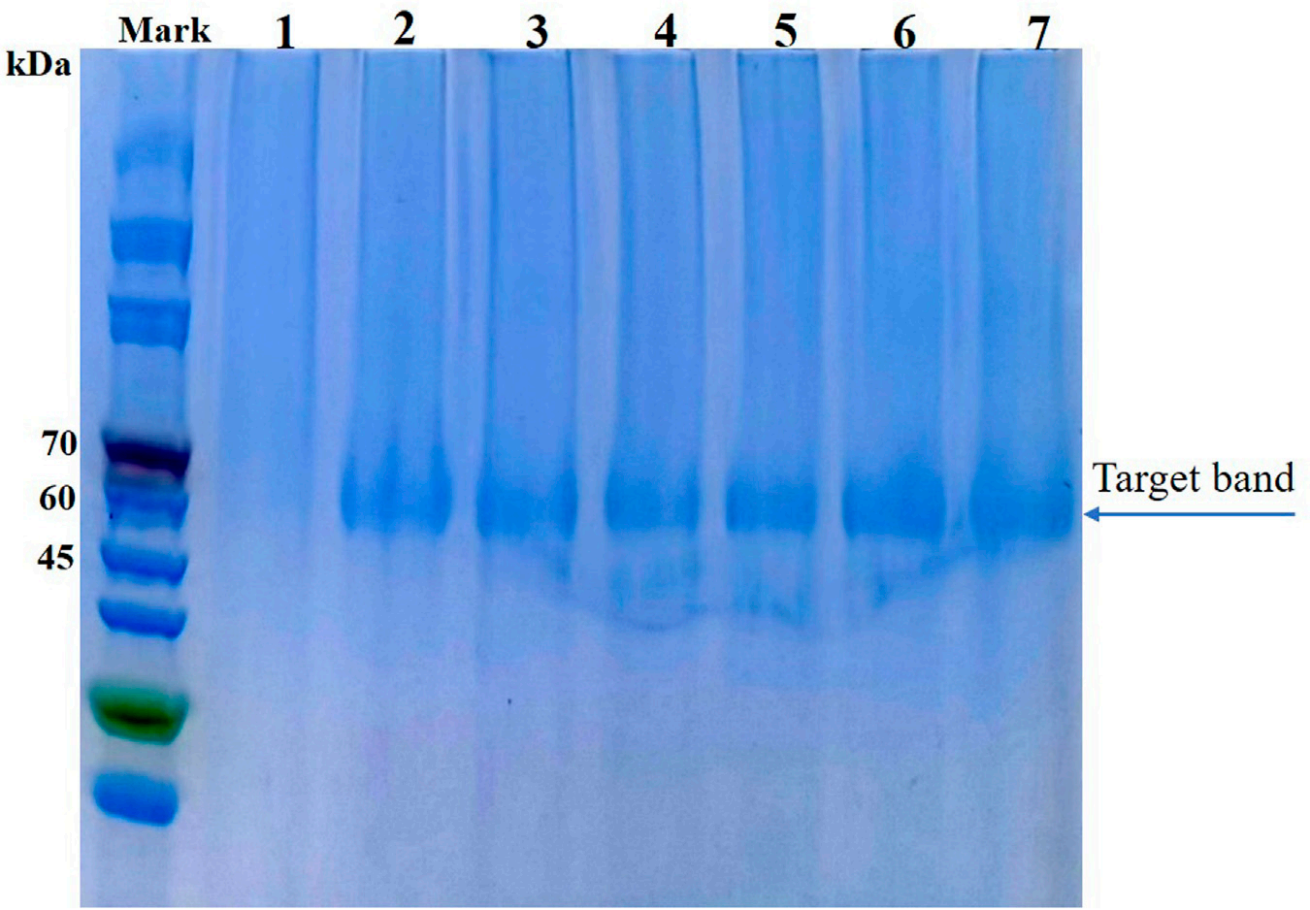
Sodium dodecyl sulfate–polyacrylamide gel electrophoresis analysis of parent and thermostable variants expressed in *P. pastoris* (analysis was performed using culture supernatant). Lane 1: Empty vector; Lane 2: Parental phytase A1; Lane 3: D7; Lane 4: E3; Lane 5: F8; Lane 6: F9; Lane 7: G10.

Enzyme yield and properties can be affected by the expression host (Muller et al., 1998). Lee et al. compared the performances of various hosts, including *Saccharomyces cerevisiae*, *Schizosaccharomyces pombe* and *P. pastoris*, for the expression of AppA2 phytase; they found that the *P. pastoris* system produced the highest enzyme activity (Lee et al., 2005). The AppA phytase derived from *Citrobacter amalonaticus* and expressed by *P. pastoris* had a protein yield of 0.42 g/L and enzyme activity of up to 15,000 U/mL (Luo et al., 2007). These results indicate that *P. pastoris* is an efficient expression system, especially for heterologous expression of phytases. In the present study, parental phytase and the thermostable variants D7, E3, F8, F9, and G10 were expressed in *P. pastoris* KM71H for purification and characterization. The gene encoding phytase without its native signal sequence was cloned and inserted into the pPICZα expression plasmid to generate an in-frame fusion with the yeast α-factor under the control of the AOX1 promoter. The resulting recombinant plasmid was transformed into *P. pastoris*; enzyme activity and yield in the supernatant were analyzed after methanol induction for 72 h in shaker flask culture. All variants showed elevated enzyme activities compared to parent; E3, G10, F8, and D7 showed increases of 1.37-fold, 1.40-fold, 1.43-fold, and 1.64-fold, respectively; while the enzyme yield of mutants D7, E3 and F8 are significantly lower than parent phytase (*p*-value < 0.01), the enzyme yield of mutants F9 and G10 are significantly higher than parent (*p*-value < 0.01) (Table 2). The expression levels of parent phytase and variants were analyzed through sodium dodecyl sulfate–polyacrylamide gel electrophoresis (Figure 2). As shown in Figure 2, a specific protein band was observed for samples that contained parent or variant phytases (lanes 2–7), while no band was observed for the negative control sample (lane 1, empty vector). The molecular weight of each band was approximately 60 kDa, which was higher than the theoretical molecular weight (44.9 kDa); this finding indicated that these proteins might be affected by glycosylate modification. No other secreted protein bands were found in any samples, as shown in Figure 2. This specificity reflects the benefit of using *P. pastoris* as a phytase expression host; it allows tedious protein purification steps to be avoided in industrial applications.

**TABLE 2:** The Enzyme activity and yield of parent and mutants expressed in *P. pastoris*

Strain	Parent	D7	E3	F8	F9	G10
Enzyme activity (U/mL)	508.6 ± 21.5	837.2 ± 17.8	698.6 ± 50.6	727.6 ± 43.2	636.2 ± 34.4	713.2 ± 52.8
Enzyme yield (mg/mL)	0.06 ± 0.003	0.04 ± 0.002	0.04 ± 0.004	0.04 ± 0.005	0.08 ± 0.01	0.08 ± 0.002

### 3.3 Enzyme purification and characterization

**FIGURE 3:**
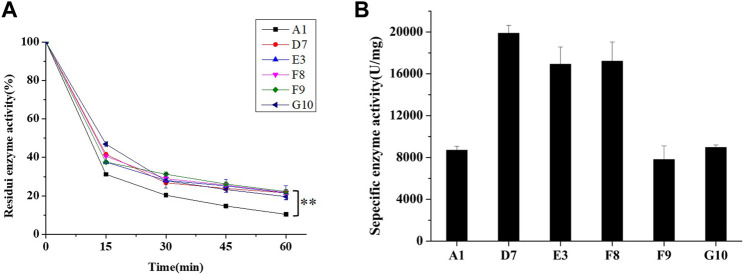
Characteristics of parent and mutants expressed in *P. pastoris*. **(A)** Residual activities of parent and mutants after treatment at 99°C for 60 min. **(B)** Specific enzyme activities of parent and mutants (determined at 37°C, pH 5.5). For statistical analysis, *t*-test was performed to compare to wild-type (A1) after treatment at 99°C for 60 min, asterisks represent significance: **, *p* < 0.01; *n* = 3.

For analysis of their thermal stabilities in extreme high-temperature environments, the phytases secreted by *P. pastoris* were purified using Ni-nitriloacetic acid metal affinity chromatography; the thermostability of each purified phytase was determined by measuring residual activity after treatment at 99°C for various lengths of time. After they had been heated to 99°C for 60 min, mutants D7, E3, F8, F9, and G10 showed improvements of 10.6%, 11.5%, 11.8%, 12.2%, and 9.6% in thermostability, respectively, compared to parent (Figure 3A). In addition, we found that phytases expressed in *P. pastoris* had significantly greater thermostability than did phytases expressed in *E. coli*; this improved thermostability was observed for both parent and mutant. The half-life of parent expressed by *E. coli* was 1.5 min at 80°C, while the half-life of parent expressed by *P. pastoris* was 5 min at 99°C. Previous studies have suggested that extensive glycosylation in *S. cerevisiae* causes CbAppA phytase to exhibit significantly greater thermostability (Kim et al., 2006). Therefore, the glycosylation modification in *P. pastoris* may improve the thermostability of *E. coli* phytase. This finding indicates that *P. pastoris* is advantageous because of its cost-effectiveness and high expression level of *E. coli* phytase, along with its post-translational modification, folding, and secretion effects during eukaryotic protein expression; therefore, *P. pastoris* is an ideal expression host for phytase.

During protein engineering, improvements in the thermostability of mutant proteins often occur at the expense of specific activity and catalytic efficiency (Han et al., 2019; Xing et al., 2021). Consequently, the specific activities of purified phytases were assessed. As shown in Figure 3B, the specific activity of A1 (parent) was 8509.27 ± 371.19 U/mg, which did not significantly differ from the specific activity of G10 (8964.76 ± 230.5 U/mg) but was slightly higher than the specific activity of F9 (7806.78 ± 1394.79 U/mg). The specific enzyme activities of D7, E3 and F8 were 19,881.68 ± 761.98 U/mg, 16920.76 ± 1651.58 U/mg, and 17211.71 ± 1832.80 U/mg, respectively; these were 2.33-fold, 1.98-fold, and 2.02-fold higher than the specific activity of A1. Thus, the improved thermostabilities of D7, E3, and F8 did not compromise their specific activities. Accordingly, D7, E3, and F8 were selected for kinetic analysis using phytate as the substrate at pH 5.5°C and 37°C. As shown in Table 3, the catalytic efficiency (***kcat/Km***) values of mutants D7, E3, and F8 were significantly greater than the catalytic efficiency value of the parent (*p* < 0.05); the respective improvements were approximately 79.8%, 73.2%, and 92.6%, respectively. The *K*_*m*_ values of D7 and E3 were 1.18 ± 0.22 and 1.16 ± 0.11 mM, respectively; both were slightly higher than the *K*_*m*_ value of the parent (0.91 ± 0.12). The *K*_*m*_ value of F8 was 0.87 ± 0.66 mM, which was lower than the *K*_*m*_ value of the parent; this indicated higher affinity for binding to the substrate. In general, D7, E3, and F8 exhibited improved thermostability and increased catalytic activity; in particular, F8 showed a stronger affinity for substrates, compared with the affinities of other phytases.

**TABLE 3:** Kinetics of parent and D7, E3, F8^a^.

Phytase	*K*_*m*_ (mM)	V_max_ (μmol min^-1^ μg^-1^)	*K*_*cat*_ (S^−1^)	*kcat/Km* (S^−1^ mM^-1^)
A1	0.91 ± 0.12	32.51 ± 1.50	24,332.0 ± 1126.2	26,795.3 ± 3513.4
D7	1.18 ± 0.22	75.55 ± 6.43	56,542.4 ± 5900.9	48,196.6 ± 3989.3
E3	1.16 ± 0.11	72.14 ± 4.68	53,991.6 ± 3503.1	46,424.1 ± 1615.5
F8	0.87 ± 0.66	60.28 ± 2.48	45,115.6 ± 7871.6	51,612.6 ± 2498.4

^a^ Kinetics was determined at 37°C, pH 5.5.

### 3.4 Saturation mutation of C75 and structural analysis of mutant phytases

**FIGURE 4:**
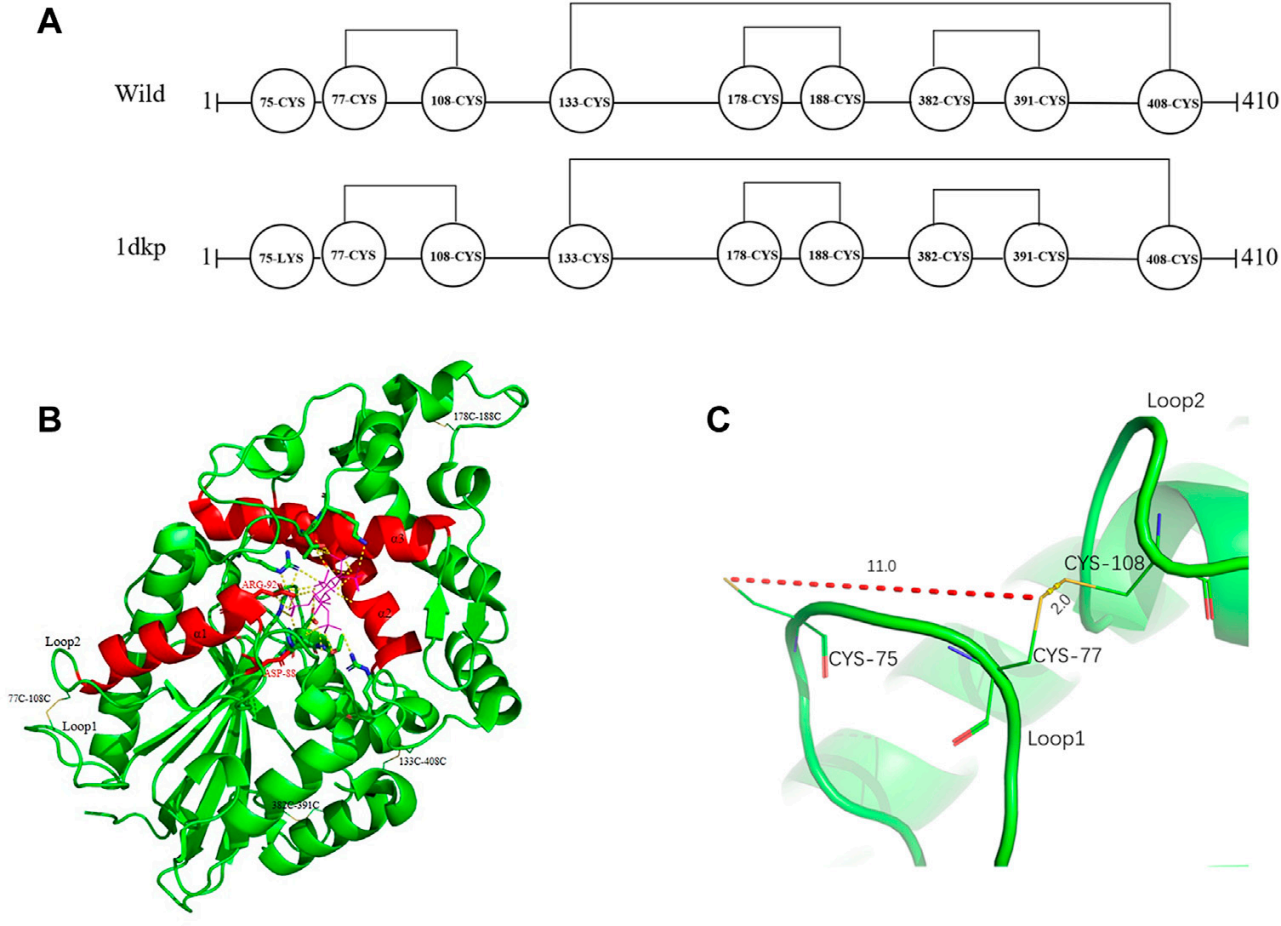
Distribution of disulfide bonds in *E. coli* phytase and their effects on phytase thermostability. **(A)** Diagram of the distribution of disulfide bonds in *E. coli* phytase. **(B)** Overall structure of phytase, with disulfide bonds in yellow and the active pocket in red. **(C)** Formation of the C77–C108 disulfide bond and nearby residues.

**FIGURE 5:**
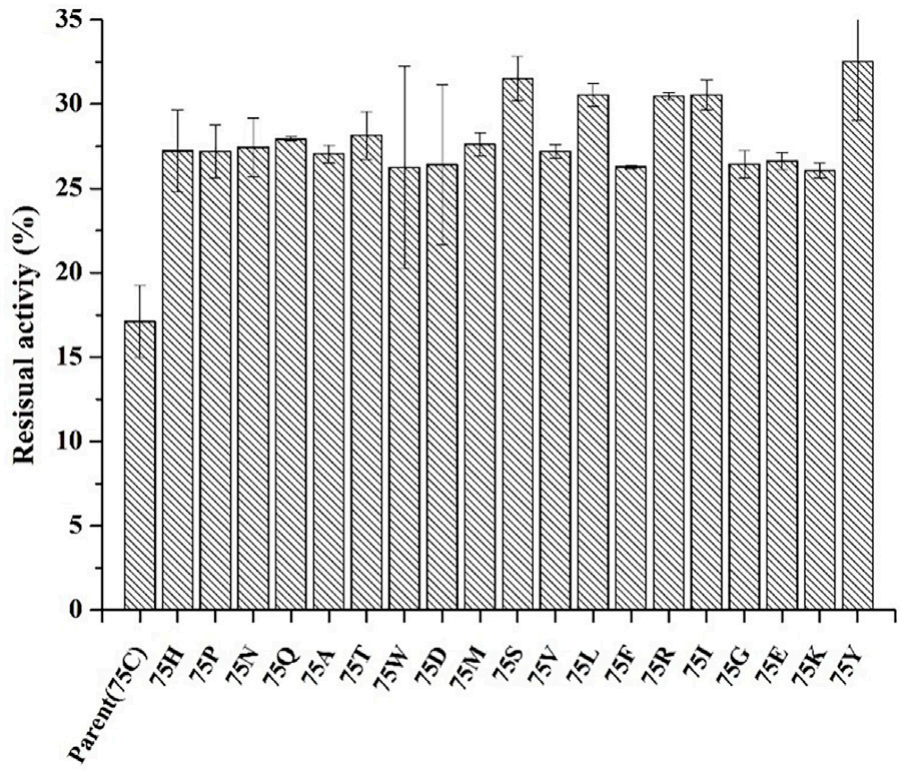
The residual enzyme activity of mutants after saturation mutation at position 75 of phytase. (incubating at 99°C for 30 min).

**FIGURE 6:**
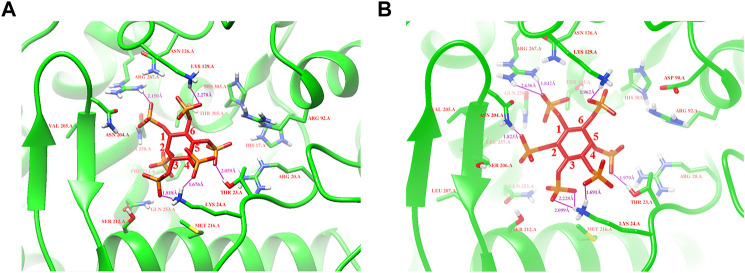
Modeling of parent and mutant F8 with phytate. **(A)** The binding position of phytate in the active-site cleft of parent; **(B)** The binding position of phytate in the active-site cleft of F8. Hydrogen bonds are represented by purple solid dash, substrate phytate is represented by a red stick.

Based on protein structures and catalytic properties, phytases can be divided into four classes: histidine acid phosphatases, protein tyrosine phosphatase-like phytases, β-propeller phytases and purple acid phosphatases. *E. coli* phytase is a member of the histidine acid phosphatase family isolated from the periplasm of *E. coli*; this enzyme is highly specific for phytate and has the highest specific activity of any characterized phytase (Mrudula Vasudevan et al., 2019). To elucidate the contributions of substituted residues to the thermostabilities of mutant enzymes, the tertiary structures of parent and mutant were modeled based on the reported structure of phytase (Protein Data Bank ID: 1DKP) from *E. coli* (Lim et al., 2000). The structure shows that the α1-helices, α2-helices and α3-helices from α-domain partially define the substrate binding region with a number of helices and loops packed around them and contains a conserved sequence motif RHGXRXP in the active site (Figure 4B). Based on Table 1, all thermostable mutants of phytase have substitution mutations at the C75 residue, indicating its crucial role in maintaining thermostability. To investigate this further, a site saturation mutagenesis study was performed on C75 and the resulting variants were expressed in *P. pastoris*. As shown in Figure 5, the phytase residual enzyme activities were all increased when the C75 was substituted with any of the other 19 residues. Notably, variants C75S and C75Y exhibited the highest residual enzyme activity and were the substitution residues of mutants E3 and F8, respectively, confirming that the improvement of the thermostability of E3 and F8 are due to the substitution of the cysteine residue at position 75. The 3D structure of phytase shows that C75 residue is located in the loop1 region, away from the active pocket (Figure 4B). To elucidate the mechanisms by C75 residue substitution contributes to thermostability, the intramolecular interactions of thermostable mutant (F8) associated with residue substitutions were investigated using the Protein Interactions Calculator online tool (http://pic.mbu.iisc.ernet.in/) (Tina et al., 2007). In the vicinity of residues 75, we observed no changes in factors such as hydrogen bonds, salt bridges, or hydrophobic interactions that affect protein stability. Therefore, we speculate that the substitution of C75 may affect the overall structure of the phytase. Subsequently, we performed Molecular docking analysis s of parent and the mutant (F8) and substrate phytate using Autodock Tool (Figure 6). The binding energy between the mutant F8 docking and structures phytate was −4.24 Kcal/mol, which is much lower than that of the parent (−2.07 Kcal/mol). This indicated that the mutant F8 is more likely to bind to phytate after substitution of C at the 75 site. Analyzing the binding position of phytate in the active-pocket cleft in the docked structure, the parent docking structure shows that five hydrogen bonds were formed in active pocket, 1-phosphate-Arg267, 3-phosphate-Lys24, 4-phosphate-Lys24, 5-phosphate-Thr23 and 6-phosphate-Arg129 (Figure 6A), the docking structure of F8 showed that except for these five hydrogen bonds, three additional hydrogen bonds were formed, 1-phosphate-Arg267, 2-phosphate-Asn204 and 3-phosphate-Lys24 (Figure 6B). These three additional hydrogen bonds make the binding of F8 to the substrate more stable, which explains the higher specific activity and catalytic efficiency of mutants after the substitution of C75 residues.

### 3.5 Effect of C77-108 disulfide bonds on the thermostability of *E. coli* phytase

Disulfide bonds are presumed to reduce conformational entropy in the unfolded state, thereby increasing the stability of the folded protein against many extracellular and secreted proteins (Zhang et al., 1994). Among the histidine acid phosphatase-superfamily phytases for which crystal structures have been resolved thus far, all have been found to contain three to five native disulfide bonds (Chen et al., 2015). For example, PhyB from *A. niger* contains 5 disulfide bonds (Kostrewa et al., 1999), phytase from *Debaryomyces castellii* contains 3 disulfide bonds (Ragon et al., 2009), and AppA phytase (in this study) from *E. coli* contains 4 disulfide bonds, including three consecutive (C77–C108, C178–C188 and C382–C391) and one non-consecutive disulfide bond (C133–C408) (Figure 4A). Sequence alignment analysis indicated that the parental phytase used in this study shares 97% homology with 1DKP, but there is a crucial difference: the residue at position 75, which originally was lysine, has been altered to cysteine (Figure 4A). The cysteine residue at position 75 (C75) is located near the disulfide pair C77–C108 (Figures 4A, C). Our saturation mutation study of the C75 residue demonstrated that this alteration resulted in reduced stability of the phytase (Figure 5). Based on these findings, we initially theorized that the presence of cysteine at position 75 might lead to the formation of new disulfide bonds between C75 and C77, which could disrupt the original disulfide bond between C77 and C108. The C77–C108 disulfide bridge spans both loop 1 and loop 2 regions (Figure 4C). Disulfide bond engineering research has demonstrated that stabilizing disulfides are typically distributed in flexible loop regions (Dani et al., 2003). As a result, the native disulfide bond formed between C77 and C108 is likely more favorable for maintaining structural stability, compared to the bond between C75 and C77.

**FIGURE 7:**
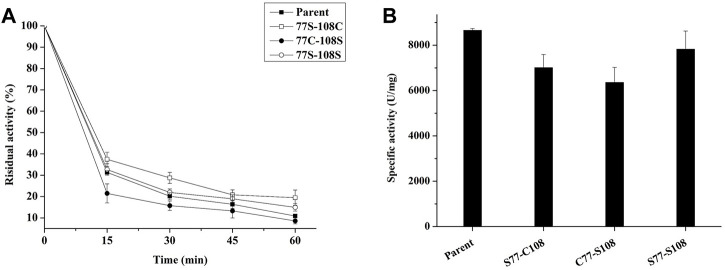
Effect of C77-C108C disulfide bonds on the thermal stability of phytase. **(A)** The thermostability of mutants with each pair of cysteine residues involved in disulfide bond formation mutated to serine (incubating at 99°C for different times); **(B)** The specific enzyme activity of mutants with each pair of cysteine residues involved in disulfide bond formation mutated to serine.

To test our hypothesis, we employed site-directed mutagenesis to remove the natural disulfide bond pair C77–C108, and then substituted C77 with S77 (S77–C108), C108 with S108 (C77–S108), and finally C77–C108 with S77-S108. The resulting mutants were expressed in *P. pastoris* and their thermostability was evaluated through incubation at 99°C for various time periods. As seen in Figure 7B, the removal of the disulfide decreased the specific enzyme activity of all mutants, however, the thermal stability of mutants S77–C108 and S77–S108 was enhanced, with residual enzyme activities of 19.5% and 14.9% after treatment at 99°C for 60 min, respectively, was higher than the 10.8% observed in the parent (Figure 7A). The results suggests that the natural disulfide bonds of C77–C108 may not enhance the thermal stability of *E. coli* phytase within the HAPs family. This finding challenges the conventional view that disulfide bonds are contributing to the thermal stability of *E. coli* phytase (Lim et al., 2000), and provides a new strategy for future phytase thermostability engineering.

## 4 Conclusion

In this study, we employed epPCR to improve the thermostability of an *E. coli* phytase. From approximately 19,000 clones, we identified five mutants with improved thermostability; mutants D7, E3, and F8 also showed significant increases in specific activity and catalytic efficiency. Sequence analysis of the mutants demonstrated that substitution at residue 75 is crucial for enhancing stability and catalytic efficiency. Docking structure analysis revealed that substitution of the C75 residue allowed the mutants to form additional hydrogen bonds in the active pocket, thereby facilitating binding to the substrate. In addition, we confirmed that the intrinsic 77C–108C disulfide bond in *E. coli* phytase is detrimental to its stability. Our work not only generated a number of thermostability improved phytase mutants, but also provided a theoretical basis for us to reveal the thermostability mechanism of phytase, which will facilitate the rational design of phytase in the future.

## Data Availability

The original contributions presented in the study are included in the article/Supplementary material, further inquiries can be directed to the corresponding author.
