# Supplementary material for: Thermostability enhancement of Escherichia coli phytase by error-prone polymerase chain reaction (epPCR) and site-directed mutagenesis
Source: Front Bioeng Biotechnol. 2023 Mar 30;11:1167530. doi: 10.3389/fbioe.2023.1167530 (PMC10101328; doi:10.3389/fbioe.2023.1167530)
Supplement: Supplementary file 1 [file Table1.DOCX]

**Thermostability enhancement of *Escherichia coli* phytase by error-prone polymerase chain reaction (epPCR) and site-directed mutagenesis**

Hongguan Xing ^1,2^, Pingping Wang^2^, Xing Yan^2^, Yi Yang ^1^, Xinliang Li ^3^, Rui Liu ^3^ Zhihua Zhou^1,2*^

^1^ School of Pharmacy, East China University of Science and Technology, Meilong Rd 130, Shanghai, 200237, China

^2^ CAS-Key Laboratory of Synthetic Biology, CAS Center for Excellence in Molecular Plant Sciences, Institute of Plant Physiology and Ecology, Chinese Academy of Sciences, Shanghai 200032, China

^3^ CJ, Youtell (Shanghai) Biotech Co., Ltd, Ste 302, Bldg 7, 526 Ruiqing Rd, Shanghai 201201, China

*Correspondence to Zhihua Zhou, Email: zhouzhihua@sippe.ac.cn

Tel: +862154924050

Fax: +862154924049


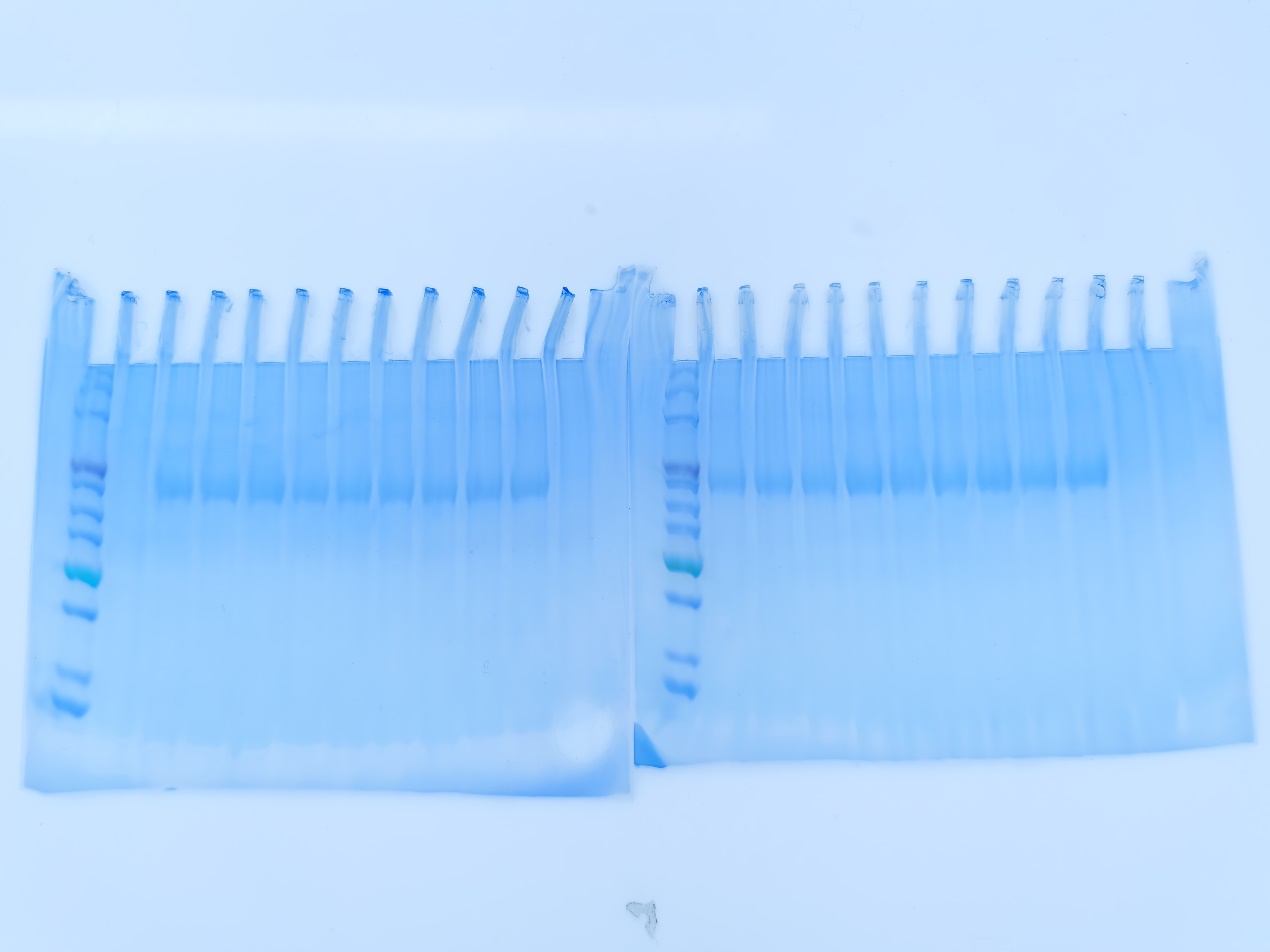


Fig. S1 Sodium dodecyl sulfate–polyacrylamide gel electrophoresis analysis of parent and thermostable variants expressed in *P. pastoris* (analysis was performed using culture supernatant). Left, Lane 1: Mark; Lane 2: Empty vector; Lane 3-5: Parental phytase A1; Lane 6-8: D7; Lane 9-11: E3; Right, Lan1: Mark; Lane 2-4: F8; Lane 5-7: F9; Lane 8-10: G10.
